# Development of flagella bio-templated nanomaterials for electronics

**DOI:** 10.1186/s40580-014-0010-x

**Published:** 2014-03-21

**Authors:** Wonjin Jo, U Kei Cheang, Min Jun Kim

**Affiliations:** 1 Institute for Multidisciplinary Convergence of Matter, Korea Institute of Science and Technology, 136-791 Seoul, Republic of Korea; 2 Department of Mechanical Engineering & Mechanics, Drexel University, 3141 Chestnut Street, Philadelphia, PA 19104 USA

**Keywords:** Flagella, Bio-template, Nanomaterial, Dye-sensitized solar cell, Lithium ion battery, Electronic

## Abstract

Bacterial flagella with their unique structural properties have proven to be promising bio-templates and can be exploited for the creation of nanomaterial with very high aspect ratio and surface area. Their chemically modifiable surfaces allow the flagella be modified to possess electrical/electronic properties. Their extraordinary physical properties along with the many possibilities for manipulation make them ideal systems to study for the purpose of developing nanoelectronics. First, this article reviews the characteristics of bacterial flagella and their utilization as biologically inspired templates. Next, the use of bio-templates for electronic systems such as dye-sensitized solar cell and lithium ion battery is discussed. Finally, we show the future directions for the use of flagella biotemplatednanomaterials for applications in electrical engineering fields.

## Background

The transition to renewable energy is met with the growing demand to develop new generations of electronic devices with improved efficiency and environmental performance in order to address the concerns regarding limited energy supplies and environmental issues. Moreover, the challenges in manufacturing extremely small features on mass-produced multifunctional devices incite the need to search for novel methods for the synthesis of the small scaled material integrated with high performance of functionality. As a result, the interface between nanosystems and biosystems is emerging as one of the largest and most active areas of technology to address the requirements and specifications for developing renewable energy [1,2]. The combination of bio and nano hold the promise to yield advances in the creation of new and powerful methods that enable direct, sensitive, and rapid energy generation [3,4]. In this respect, devices using on nanomaterials synthesized by biologically inspired templates can be one of the most promising platforms for direct ultrasensitive electrical detection and for building functional interfaces of core components [5]. Biological templates have self-assembled hierarchical structures and can be massively obtained in nature, allowing the massive fabrication of nanostructured materials with precise dimensions and structures. Furthermore, biologically templated nanomaterial can be easily modified to change their natural functions and properties to create new types of highly efficient electronic devices, such as dye-sensitized solar cells (DSSCs) or lithium ion batteries (LIBs), with lightweight and high performance.

Using bacterial flagella as biologically inspired templates is a relatively new approach to assemble nanoscale architectures, which can revolutionize the fabrication process and the materials used in ultra-densely integrated electronic devices. Of particular significance is the finding that it is possible to selectively metallize the flagella templates or flagella based templates to obtain electrical properties through interelement positioning at the nanometer scale [6,7]. However, even though their natural tubular nanostructure and the ease manipulation of their surface properties have already been well demonstrated, there is a lack of progress in the field of flagella-based device fabrication. To stimulate advances in using flagella as bio-templates in these forward-looking fields, this article will examine three main sections. The first section explains the characteristics of bacterial flagella to highlight their attractive properties that can be exploited for nanofabrication. Next, this article will introduce current electronic devices, including DSSCs and LIBs, built using other bio-templates such as virus or diatom. In the last section, we will discuss recent developments in the flagella based bio-templates and present the future prospects of applying them in the electrical areas.

### Characteristics of bacterial flagella

Bacterial flagella, such as those from *Escherichia coli* (*E. coli*) or *Salmonella typhimurium* (*S. typhimurium)*, are organelles used for propulsion. Bacterial flagella are self-assembled helical nanostructures, approximately 20 nm in diameter and 10 μm in length. The flagella have extraordinary mechanical properties; they are extremely stiff and have an elastic modulus estimated to be around 10^10^ N/m^2^ [8–10]. As a result, they have remarkable durability and stability. In addition, they have the properties to be active nanostructures. They are able to actively adapt to the environment by changing helical handedness and pitch in response to various external stimuli; this is known as polymorphic transformation. Specifically, bacterial flagella can undergo polymorphic transformations both in loaded and unloaded conditions due to chemical, electrical, thermal, mechanical, or optical stimulation. Furthermore, flagellar filaments’ remarkable durability and stability allow them to withstand high temperature (up to 60°C) and extreme pH (7 ± 4) [11–13].

### Structure of flagellar filaments

**Figure 1:**
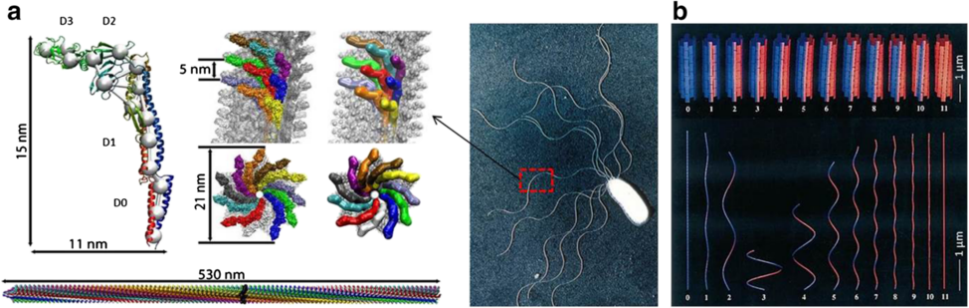
**Structure and polymorphic transformation of flagellar filaments.** **(a)** Single flagellin monomer and arrangement of the monomers in the filament [20]. Reprinted with permission from ref. 20, Copyright © 2006, Elsevier. **(b)** Bacterial flagellar filament polymorphic forms, shown in a curvature vs. twist plot. There are two straight forms, L (Left) and R (Right), and 10 helical forms. Of the named helical forms, normal and coiled are left-handed and the others are right-handed [21,22]. Reprinted with permission from ref. 21, Copyright © 1998, Elsevier.

The flagellar filaments are polymers consisting of a single type of protein called flagellin, which have a molecular weight of about 55,000 Da, an outer diameter of 120–250 Å, and an inner diameter of 20 Å [14]. They are devoid of the amino acids cysteine or tryptophan and of any known enzymatic activity [15]. The flagellin protein is made up of four domains: D0, D1, D2, and D3 (Figure 1a). Of the four domains, D0 and D1 are essential for the assembly of the filament and for the change of conformation leading to polymorphic transformation [16]. The D3 domain is responsible for the antigenic response of bacteria [17]. As mentioned, the flagella can take on a number of polymorphic configurations. A filament with normal configuration is left-handed with a helical pitch of about 2.2 μm, a helical diameter of about 0.4 μm, and 5340 flagellin subunits per turn. The surface lattice of the filament is hexagonal, and all the subunits can be traced with 1-start, 5-start, 6-start, or 11-start helices. The 11-start helices (protofilaments) are nearly longitudinal. The filaments are helical because the subunits can bond in two different ways, with each protofilament forming a cooperative unit. Filaments with only one type of protofilament are straight. In L-type straight filaments the 11-start helices twist about 2.5° to the left, while in R-type straight filaments twist about 7° to the right. The intersubunit distance is 52.5 Å (long) for the L-type and 51.8 Å (short) for the R-type. It is thought that all the flagellins in one protofilament are of only one-bonding type, forming either a long or short protofilament. The mechanical strain in a filament is minimized if protofilaments of the same type are adjacent to one another [18,19]. This leads to the prediction of 12 polymorphic forms, 2 straight, and 10 helical, as shown in Figure 1b.

### Polymorphic transformation of flagellar filaments

Given their structural makeup, filaments of different polymorphic forms can be obtained by using different flagellin types from different bacterial strains or by mixing different quantities of flagellin obtained from L-type and R-type straight filaments [23]. However, a filament’s polymorphic configuration depends not only on the flagellin type, it also depends upon environmental conditions (i.e. pH, ionic strength, and temperature) as established in a series of experiments by Kamiya and Asakura [11,12,24]. Most of these experiments were performed with wild-type strains of *Salmonella*, but identical results were obtained with an *E. coli* K12 strain [25]. As an example, filaments in 0.1 M KCl buffer expose to a raise in pH from 7 to 9 changes from the normal form to the coil form, which consequentially reduces their end-to-end length by about a factor of nearly 3. The flagellar filament also have an induced dipole moment of 5 × 10^-24^C⋅m in an electric field of E = 10^6^ V/m [26]. For example, straight polymorphic filaments align along the field, but close-coiled forms align with the helical axis perpendicular to the field [26]. Under the appropriate condition, the filaments can even go so far as to change the handedness of the flagellar helix. Such astonishing degree of actuation opens up the possibility to utilize flagellar filaments for a broad range of applications.

### Flagella depolymerization and repolymerization

**Figure 2:**
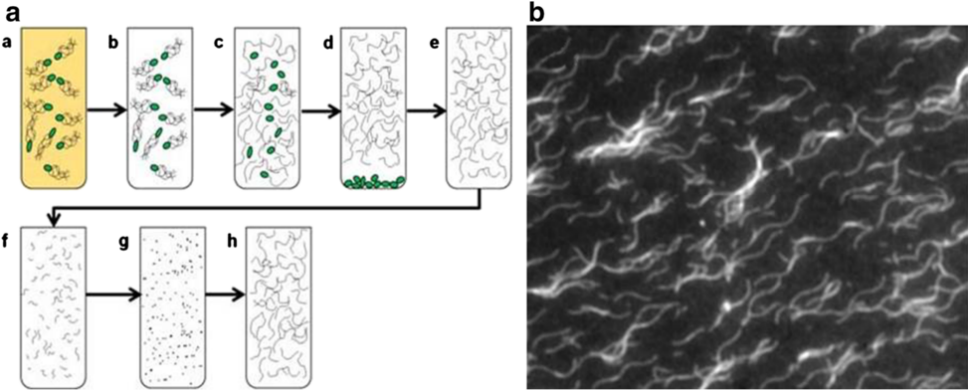
**Flagella depolyerization and repolymerization procedures.** (Left) Method for the preparation of flagellar filaments; (a) bacteria are grown for 10 hours in LB broth; (b) centrifuged and resuspended in PBS; (c) vortexed to separate flagellar filaments; (d) bacterial cell bodies are pelleted by ultracentrifugation; (e) the supernant is resuspended in PBS; (f) the flagellar filaments are broken into smaller pieces by sonication; (g) depolymerized by heating; (h) the seeds are added to the solution of flagellin and repolymerization is carried out for 24 hours. (Right) Scanning electron microscopy (SEM) images of flagellar filaments repolymerized from S. typhimurium. Reprinted with permission from ref. 7, Copyright © 2009, Elsevier.

The length of the flagellar filaments can be modulated using depolymerization and repolymerization processes as shown in Figure 2a; in other word, flagella can be broken down into monomers (depolymerization) and then reassembled (repolymerization) with the desired length [7,27]. This can be achieving in an in vitro stepwise procedure where the parameters of the process can be manipulated in order to control the length of the flagella. First, the flagella filaments are harvested from bacterial cells in a saturated culture by shearing the filaments off from the cells using a vortex. The filaments are then isolated form the cell bodies by differential centrifugation. A portion of the isolated flagella is sonicated to form short segments of flagella, which will be used later as seeds to initiate repolymerization, while the rest is heated to 65°C for complete depolymerization to flagellin. Next, the flagella are prepped for repolymerization by adding the flagella seeds to the flagellin solution. The flagella are left to repolymerize overnight. These filaments grow uni-directionally from one end of the seed, so it is straightforward to construct simple block copolymers by changing the types of flagellin in the supernatant fraction of the solution. When the filaments are first detached from cells by vortex, they are generally shorter than 10 μm with a broad distribution of length. However, they can be repolymerized to give a majority in the range of 10 – 25 μm, with some as long as 75 μm [22]. Once made, they can be stored for months in the polymerization buffer. Figure 2b shows optical micrograph of fluorescently-labeled flagellar filaments repolymerized from *S. typhimurium.*

### Biomaterials as templates for the application of electronics

Recently, there is a fast growing interest in biological template technology, such as the use of bacteria [28,29], virus [30–32], diatoms [33,34], butterfly wings [35,36], and so on. Most biological species possess a variety of unique characteristics such as distinctive nano or microstructures. Thus, it is very desirable to utilize these naturally formed unique structures as templates to create complex inorganic structures for different applications, such as nanoelectronics. Biotemplating is an eloquent and economic way to massively synthesize hierarchically periodic micro and nanostructures for electrical parts without using complex and expensive processes, making the biotemplate technology ideal for the synthesis of high surface area nanomaterials. In this section, we will review some recent works on DSSC and LIB devices built using biotemplate based nanomaterials.

### Dye-Sensitized Solar Cell (DSSC)

The negative impacts from existing energy source, such as environmental impacts, and the increasing scarcity of energy resources have prompted the need for cheap, renewable, and clean energy technologies. Solar energy has been a major focus of modern science and engineering, especially with the threat of global warming and fossil fuel depletion. Each year, 3 × 10^24^ Joules of energy radiates from the sun to the earth [37]; this manifested the idea to harness such large amount of energy through the use of solar technologies. However, existing solar technologies are expensive when compared with hydrocarbon fuels, which their limits widespread commercialization. In order to increase the appeal of solar cells, their efficient must be increased and their cost must be decreased. Over the last decade, a type of photovoltaic device called the dye-sensitized solar cell (DSSC) had emerged as a technology that can achieve reasonably high efficiency and low manufacturing cost. Unlike conventional p-n junction diode based photovoltaics, the DSSCs create photovoltaic effect by photoexcitation of dye molecules that then inject electrons into wide band gap semiconductors that are not themselves able to be photoexcited. The performance of the DSSCs can be enhanced by optimizing the structure of the nanoparticle layers (TiO_2_ or ZnO) in the photoanode, the photosensitizing dye, and the electrolyte. In a typical particle based DSSC, the solar conversion efficiency is directly related to the electron percolation through the nanoparticle layers. However, the structural disorder at the contact between two crystalline NPs leads to enhanced scattering of free electrons. Therefore, it reduces electron mobility resulting in slow percolation which may lead to a decrease in efficiency [38].

Recently, much work had been performed to improve the optical path length of photoanodes by modifying surface structure or properties of photoanode [39]. Since the light harvesting efficiency is directly affected by the photoanode properties, the optimization and improvement of photoanode plays an essential role in the performance of DSSCs [40]. One approach was to replace the two dimensional flat surface with a three dimensional structure consisting of a dense array of aligned nanoscale features, such as the hierarchically self-assembled nanomaterial. The array of nanostructures created a larger surface areas compared with the flat surface; as a result, the optical path length was increased which in turn enabled rapid collection of carriers and achieved better light-accumulation effects [41]. Furthermore, the increase of the optical path length inside DSSCs increases the probability of interaction between dye molecules which is essential to yield high light absorption and to facilitate the transfer of photo-generated electrons.

**Figure 3:**
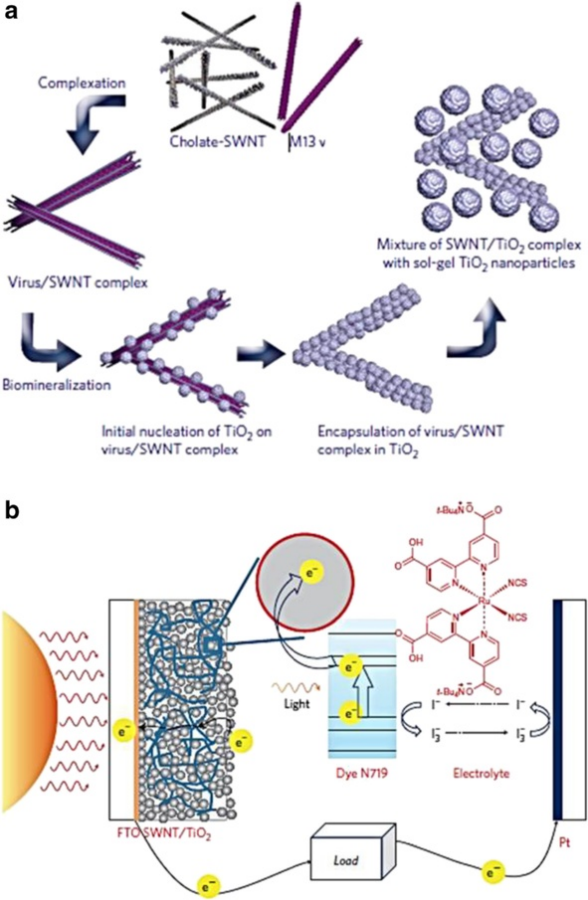
**M13 virus-based SWNT/ TiO**_**2**_ **complex was used in DSSCs.** **(a)** Schematic diagram illustrating the fabrication process of **(a)** M13 virus-based SWNT/TiO_2_ complex. **(b)** DSSCs incorporating the complex. Reprinted with permission from ref. 46, Copyright © 2011, Rights Managed by Nature Publishing Group.

**Figure 4:**
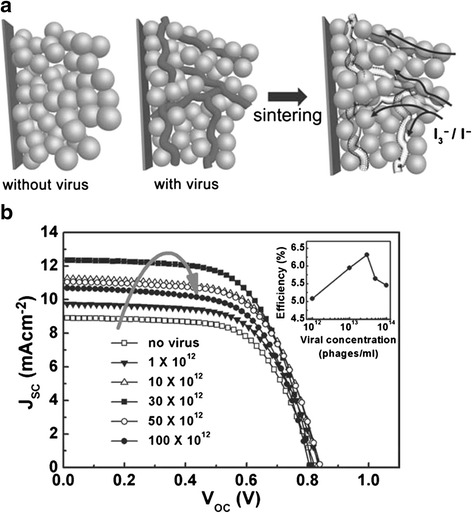
**DSSC TiO**_**2**_ **photoelectrode using M13 viruses.** **(a)** Schematic illustration of DSSC photoelectrode fabrication process shows conventional TiO_2_ nanoparticle photoelectrode and enhanced TiO_2_ photoelectrode using M13 viruses as sacrificial templates. **(b)** Current density-voltage characteristics of electrodes at various virus concentrations. Reprinted with permission from ref. 47, Copyright © 2011 WILEY-VCH Verlag GmbH & Co. KGaA, Weinheim.

To advance along the aforementioned approach, several nanotubular architectures have been investigated for the potential enhancement of electron percolation pathways, light conversion, and ion diffusion at the semiconductor-electrolyte interface [38,42,43]. Due to their lower trap density and more direct path to the current collecting electrode, arrays of nominally one-dimensional nanostructures are expected to speed up charge migration without adverse effects from recombination [44]. In an effort to further increase the power conversion efficiency, biotemplate based nanomaterials were developed in order to create paths for quick electron transfer to the conducing substrate. One such template was the M13 bacteriophage, which is a filamentous bacteriophage with a length of 860 nm and a diameter of 6.5 nm. Since the protein capsid from the M13 bacteriophage can be genetically modified with specific peptides, it allowed the formation of nanoparticles, metal oxides, or other materials [3,45]. Recently, Dang et al. integrated the single-walled carbon nanotubes (SWNTs) into photovoltaic devices by using M13 virus templates as binding agents [46]. A schematic of the fabrication process is given in Figure 3. Through the use of virus templates, SWNTs was bounded and stabilized through non-covalent binding; preserving high electron mobility by eliminating the need for surfactants and surface chemical modifications which hamper electron transfer. Additionally, the virus/SWNT complex enabled direct contact between the SWNTs and titanium dioxide (TiO_2_) which in turn improved the power conversion efficiency. In addition to their applications as a binding agent, the M13 virus was also used as a sacrificial template to create an optimized interface between the electrolyte and the TiO_2_ nanoparticles [47]; this created effective electron pathways (Figure 4a). In a typical DSSC system, uniform penetration of electrolytes is inadequate due to the randomly placed TiO_2_ nanoparticles. As a sacrificial template, the M13 virus was first used to form interconnections with the TiO_2_ nanoparticles via electrostatic binding; then the M13 virus was thermally decomposed to leave hollow channels as pathways for electrolyte penetration. Consequently, efficient electron pathways were created via the interconnected nanochannel structures and, as a result, an increased in the photo conversion efficiency was achievedover the conventional TiO_2_ photoelectrodes by 35% as shown in Figure 4b.

**Figure 5:**
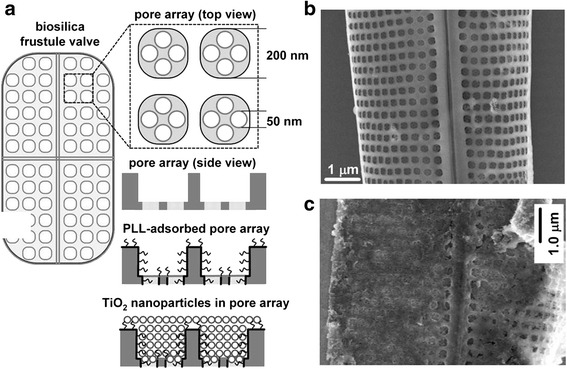
**TiO**_**2**_ **–coated diatom frusule. (a)** Scheme for the PLL-mediated deposition of TiO_2_ on the diatom frustule biosilica. **(b)** SEM image of bare diatom frustule. **(c)** SEM image of TiO_2_ –coated diatom frusule. Reprinted with permission from ref. 48, Copyright © Cambridge University Press 2011.

Diatoms, unicellular photosynthetic microorganisms, are good candidates for the DSSCs application due to their unique nanostructured patterns of silica cell walls. The size of diatoms ranges from 1 to 100 μm. The diatom silica walls could be chemically functionalized to interact with inorganic nanoparticles. Recently, Jeffryes et al. used diatom frustules, which are nanostructured amorphous silica skeleton, as templates for the deposition of nanostructured TiO_2_ (Figure 5) [33]. The process to create nanostructured TiO_2_ began with the deposition of poly-L-lysine (PLL)-TiBALDH. Next, the PLL was absorbed by the diatom silica. Finally, the PLL precipitated the soluble titanium precursor, TiBALDH, into insoluble TiO_2_, resulting in the formation of amorphous titania nanoparticles. As a result, amorphous TiO_2_ nanoparticles completely packed the frustule pores bringing about TiO_2_loading on the frustules. The resulting diatom-TiO_2_ composite material was then used in a DSSC photoanodes which effectively increased the efficiency by nearly 3 times compared with the photoanode made from only TiO_2_ nanoparticles [48].

**Figure 6:**
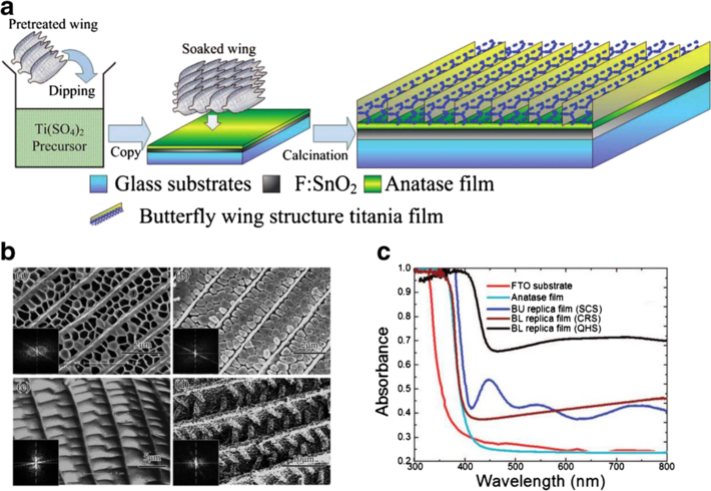
**Photoanode template using butterfly wings. (a)** Schematic illustration of the fabrication process of quasi-honeycomb structure photoanode template from butterfly wings. **(b)** FESEM images of photoanodes with different structures. **(c)** Absorption characteristics show by the wavelength-dependent absorption at the different photoanode structure. Reprinted with permission from ref. 40, Copyright © 2009, American Chemical Society.

Studies of the use of butterfly wings as the biological scaffolds for the construction of electrical devices are also noteworthy for their potential uses in DSSCs. Butterfly wings exhibit periodic submicrometer structures which can be used to create effective solar collectors [49]. The butterfly wing scales are approximately 150 μm long and ~50 μm wide; typically have highly ordered architectures with pores and layers. The wing scales’ micro features are of great importance because they can be used to create templated microstructures that can improve the light absorptivity [35,50] by increasing the surface area. Inspired by the butterfly wing scales, Zhang et al. developed a novel titania film photoanode as described in Figure 6a [40]. Three separate photoanodes with different structures were synthesized onto a fluorine-doped tin-oxide-coated glass substrate using butterfly wings as biotemplates: Quasi-honeycomb like structure (QHS), shallow concavities structure (SCS), and cross-ribbing structure (CRS). Figure 6b presents the field emission scanning electron microscopy (FESEM) images of the synthesized QHS (upper images) and SCS (below images) structures. By investigating their wavelength-dependent absorptions and current-potential curve measurements, Zhang et al. demonstrated that the QHS titania replica photoanode has an optimal light absorptivity (Figure 6c) and higher surface area, which is extremely advantageous to light harvesting and dye absorption in the DSSCs.

### Lithium Ion Battery (LIB)

To meet the demand for a clean and secure electrical energy storage unit, extensive research efforts have been made to develop rechargeable batteries with large capacity, high power, and reasonable life cycle. The most notable are the LIBs due to a number of significant advantages over competing technologies [51]. The use of lithium intercalation/alloying compounds had successfully enhanced the cycling life, safety, capacity, charge/discharge rates capability of the battery, as well as other battery characteristics such as high energy density and flexibility in shape and size. The three primary components of a LIB are the cathode, the anode, and the electrolyte. The operation of the LIB involves a electrochemical process where lithium ions migrate between the cathode and the anode while electrons flow through a close external circuit. Electrical energy is provided to the connected device via discharge where lithium ions move out of the anode (extraction) and into the cathode (intercalation). These reactions take place continuously while electrons continue to flow. Electrical energy can be restored to the battery through recharge, which is simply a reversal of the discharge process.

Though LIBs are generally smaller and lighter than other rechargeable batteries, the growing demand for portable electronics, such as cell phones and laptops, have facilitated the need to develop even smaller batteries for the propose of scaling down portable devices. However, it is still a challenge to breach the limitations of LIBs to create smaller batteries without sacrificing performance. Fortunately, the emerging technologies to fabricate and pattern nanostructures have incited a promising solution. The high surface area offered by patterned nanostructures can significantly increase the electrode/electrolyte contact areas, improved mechanical stability, and reduced distances for electron and ion transport to facilitate faster reaction kinetics. Furthermore, biogenic, bio-enabled, and bio-inspired hierarchical nanomaterials can be used to further increase reactive surface area. For instance, biological template such as viruses have proven to be extremely effect in the mass production of self-assembled hierarchically ordered structures with high surface to volume ratio. The advantages of using biotemplate technology may hold the key to a new class of electrode materials.

**Figure 7:**
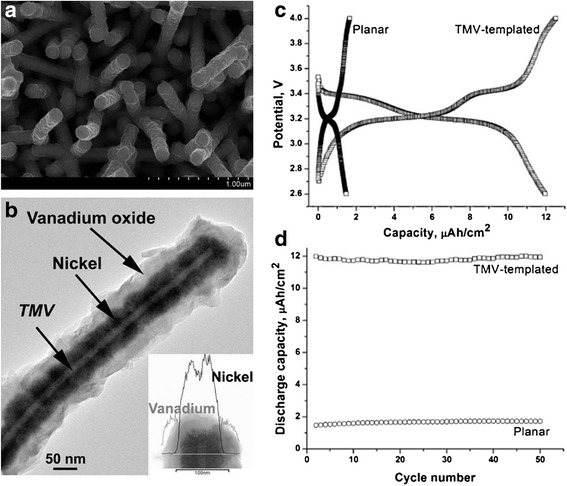
**TMV-templated V**_**2**_ **O**_**5**_ **electrodes. (a-b)** SEM and transmission electron microscopy (TEM) image of the TMV-templated Ni/V_2_O_5_ core/shell cathode. **(c)** Discharge and charge curves and **(d)** the cycling performance of the cells with TMV-templated V_2_O_5_ electrodes. Reprinted with permission from ref. 54, Copyright © 2012, Elsevier.

**Figure 8:**
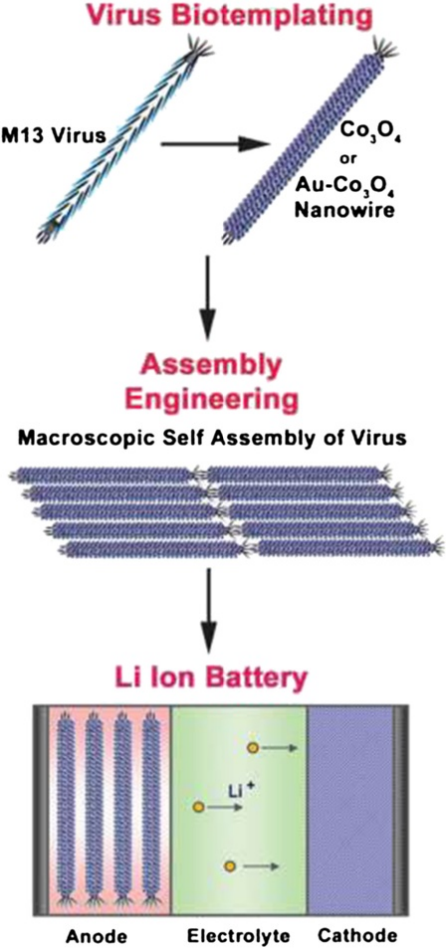
**Schematic illustration of the synthesis and assembly of M13 virus template nanowires for negative electrode materials of LIBs.** Reprinted with permission from ref. 55, Copyright © 2006, American Association for the Advancement of Science.

The tobacco mosaic viruses (TMV) are known as useful biotemplates because their size and rigid rod-like bodies which are 300 nm long and 18 nm wide and contain an internal channel of 4 nm in diameter. The biological nanostructures of viral templates with or without decoration by metal nanoparticles have been extensively studied. Royson et al. used genetically engineered TMV as robust templates for electroless deposition of nickel and cobalt [52]. The vertically patterned TMVs on a gold surface allowed for efficient and uniform metal coating, which resulted in more than 10-fold increase in available surface area. Evidently, the virus-templated electrode surfaces in a battery system showed enhanced electrode capacity, electrode life, and voltage output. Chen et al. also developed genetically engineered TMV templates to form a high capacity silicon anode with internal nickel current collector [53]. The resultant TMV/Ni/Si anodes based on high aspect ratio nanostructured surfaces produced high capacities, excellent cycle-life, and consistent rate capabilities. In these works, TMVs were genetically engineered to assign specific affinities to the targeted materials. Generally modified TMVs, which have the cysteine proteins on their surfaces, can be easily attached onto specific metal surfaces in electroless plating solutions. This is caused by the strong covalent-like interactions between the thiol groups of the cysteine protein and the metal ions. By taking advantage of the powerful binding and stabilizing properties of TMV, vanadium oxide (V_2_O_5_), a broadly utilized cathode material, was easily deposited on the nickel-coated TMV templates using atomic layer deposition process for the enhancement of cathode performance (Figure 7a and 7b) [54]. With the TMV-templated V_2_O_5_, higher capacities were achievable through the enhanced surface area resulting in the increase of active battery material loading as shown in Figure 7c and 7d. This also led to excellent electrochemical performance including high excellent rate capability and cycling stability. Like the TMV biotemplates, the M13 viruses are another potential choice for viral templates. The M13 virus templates were used to synthesize and assemble hybrid gold-cobalt oxide nanowires for advance LIB electrodes (Figure 8) [55]. Similar to the TMV templates, the M13 viruses were genetically engineered to promote strong binding affinity to cobalt ions. The virus-mediated hybrid composites generated higher initial and reversible lithium storage capacity than the pure cobalt oxide nanowires at the same current rate.

**Figure 9:**
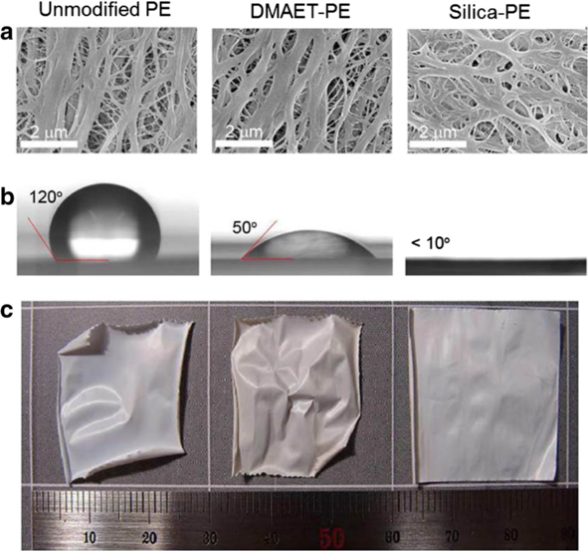
**Diatom-inspired silica coating on the surface of polyethylene (PE) separators. (a)** SEM images of separator membranes with different surface coating. **(b)** Static water contact angle measurement images shows wettability on the different separators. **(c)** Digital photographs of thermal stability of the different separators after exposure to 140°C for 1 h. Reprinted with permission from ref. 56, Copyright © 2012, American Chemical Society.

Given the importance of electrodes in battery systems, separators is also naturally a key component of LIBs since separators are directly associated with power performance and cell safety. An interesting study was performed using diatom-inspired silica coating on the surface of polyethylene (PE) separators [56]. The approach involved surface growth of a silica layer inspired by diatoms; the interfacial adhesion of the layer to the surface was inspired by mussel adhesion. The fabricated separator membranes were shown in the SEM images in Figure 9a. The silica coating increased the electrolyte wettability of the separator (Figure 9b), resulting in enhanced power, thermal shrinkage (Figure 9c), and safety.

### Flagella biological templating technology

#### Flagella bio-templated nanomaterials and metallization

Compared to the viral bio-templates, bacterial flagella templates have not been demonstrated to be used to create nanomaterials for electronic devices. However, recent promising studies had shown that flagella can be a good candidate as bio-templates. In their natural state, flagella exhibit self-assembled spiral and tubular nanostructures with extremely small outer diameter (20 nm) and controllable length. Aside from having attractive structural properties, flagella are extremely stiff and highly durable; they are stable under harsh conditions such as high temperature and extreme pH. Additionally, the solvent-exposed domains (D2 and D3) of the flagellin subunits are genetically modified, enabling simple interactions with external materials via chemical or genetic processes. In the context of fabricating biologically inspired flagella templates, some studies were also focused on using genetically engineered flagella for the well-defined functional groups on their surfaces, which led to the generation of ordered arrays of nanoparticles or monodisperse nanotubes. The peptide loop modified flagella nanotubes have been demonstrated to be useful scaffolds with high affinities for metal ions including Cu(II), Au(I), Co(II), Cd(II), and Pd(II) [57] and for the immobilization of Pd and Au nanoparticles [40,58]. Genetically modified flagella have favorable in that their surfaces can be selectively functionalized to have strong affinities with different types of nanoparticles; however, the process can be complex and require expertise.

**Figure 10:**
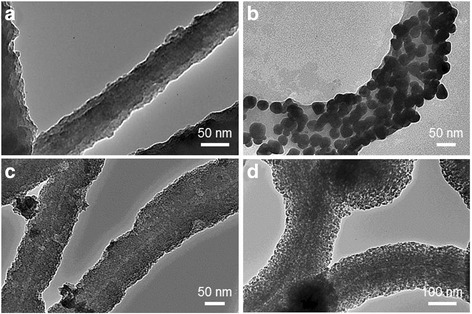
**TEM images of silica nanotubes fabricated on flagella bio-templates.** **(a)** Pristine silica nanotube and **(b-d)** metallized silica nanotubes by the deposition of gold, palladium, and iron oxide nanoparticles. Copyright © 2013 IOP Publishing (Ref. 59). Reproduced by permission of IOP Publishing. All rights reserved.

On the other hand, Kim group recently developed methods for metal oxide layer deposition on flagella surface in aqueous solution without genetic modification [7,27]. They demonstrated deposition of TiO_2_ on flagella isolated from *S. typhimurium* via a biomimetic mineralization process under the temperatures of 40°C and 50°C [7]. Also, methods were develop to utilize flagella biotemplates for fabrication of silica mineralized nanotubes [27]. The process involved hydrolysis and condensation reaction which was initiated by pretreatment of flagella with amino-propyltriethoxysilane, followed by the addition of tetraethoxysilane at room temperature. Moreover, the biologically derived silica nanotubes were improved and modified by decoration with gold, palladium, and iron oxide nanoparticles through wet chemical methods [6,59]. Form the TEM images shown in Figure 10, one can clearly see the silica nanotubes before (Figure 10a) and after the nanoparticle deposition (Figure 10b-10d). This method can fabricate nanotubes with unique and distinctive properties using fast and simple procedures, without the need for genetic modifications.

#### Characterization of electrical properties of flagella-templated nanotubes

**Figure 11:**
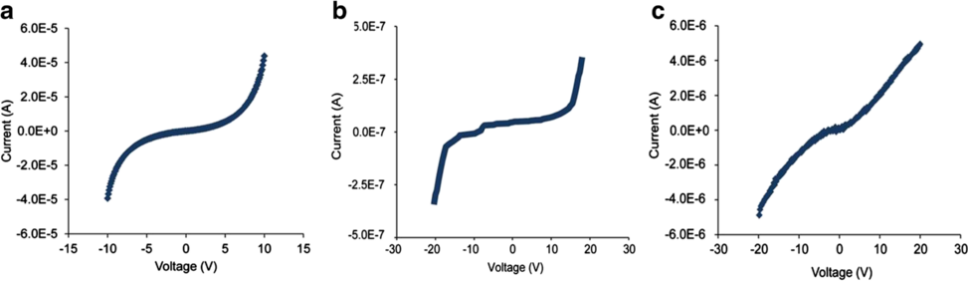
**Current–voltage characteristics of metallized silica nanotubes fabricated from flagella bio-templates.** **(a)** gold, **(b)** palladium, **(c)** iron oxide nanoparticles coated silica nanotubes. Copyright © 2013 IOP Publishing (Ref. 59). Reproduced by permission of IOP Publishing. All rights reserved.

The electrical characterization of the mineralized and metallized flagella based nanomaterials is a key step towards the realization of using flagella based nanomaterials as electrical components. Though very little is known about their energetics and electrical properties, the work done by Jo et al. have shed much light on the subject by characterizing the electrical properties of flagella-templated nanotubes [59]. Metallization, such as gold, palladium, or iron oxide nanoparticles, was explored and proven to effectively enhance the nanotubes’ electrical conductivity (Figure 11). Given the properties of the selected metal nanoparticles, the application for the metalized nanotubes can be completely different. In particular, the electrical properties of gold incite the possibility for applications in electronics, battery electrodes, and fuel cells. Given their properties, it is possible and desirable to use nanotubes fabricated via flagella biotemplates as electrical materials.

#### Flagella templated dye-sensitized solar cell

The previous results suggest the possibility that the bacterial flagella can be selected for use in the DSSCs due to their unique properties; in particular, they have controllable length, they can change their helical form through polymorphic transformation, they can be genetically modified, and their surface can be functionalized. The functionalization of the flagella also makes the positioning of flagella nanotubes on a substrate with high precision; possibly more so than positioning nanowire arrays in current DSSCs devices. The poor control over the placement of the nanowires causes the cells to not perform as well as possible. Using bottom-up and top-down nanofabrication techniques [24], it is possible to place the flagella exactly where they are desired, and highly ordered arrays can be created, forming a “*flagellar forest*”. The flagella of *S. typhimurium* will be used in the DSSC design due to the well-defined and studied polymorphic forms.

#### Engineered flagella forest

**Figure 12:**
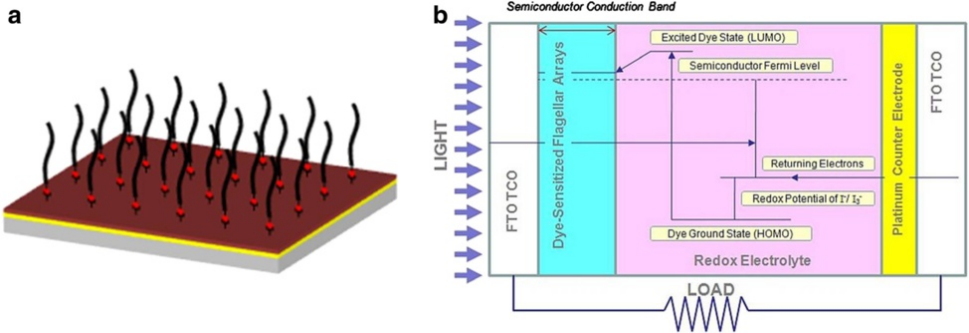
**Flagella templated DSSC. (a)** Flagellar forest assembled using avidin-biotin chemistry. **(b)** Schematic of the operation of the flagella templated DSSC.

The flagella forest is a device that harnesses arrays and patterns of flagella filaments. It consists of arrays of biotinylated flagellar filaments attached to an avidin surface treated substrate [60] in a manner where the filaments are tethered at one end. To achieve this, flagellar filaments are first functionalized at one end through biotinylation of flagella seeds followed by polymerization using non-functionalized flagellin [61]. Finally, the one-ended biotinylated flagella are introduced onto an avidinated substrate to create a flagella forest. This results in the construction of well-designed patterns of flagella arrays. The flagella forest design is shown on Figure 12a. To create a silica nanotube forest, the aforementioned flagella mineralization method can be used. Additionally, metallization of the silica nanotube forest can also be achieved.

#### Flagellar nanotube forest as a dye-sensitized solar cell

Once the flagellar nanotubes are harness into a nanotube forest device, they can be readily utilized to enhance DSSCs. A schematic of the flagellar nanotube enhanced DSSC is shown on Figure 12b. This device works by photoexciting dye molecules which inject electrons into semiconducting scaffolds which then transport the electrons to the transparent conducting electrode (TCO), upon which incident light enters the device. The dye chosen for this photovoltaic device should be a ruthenium-based dye, as demonstrated in a similar device [41]. The appropriate TCO to be used on this device is Fluorine doped Tin Oxide (FTO). The dye scaffolds used in this device will be silica nanotubes. The use of flagella as templates allows for high aspect ratio and high surface area of the dye scaffolds. This charge transfer mechanism shown in Figure 12b requires that the redox electrolyte be oxidized so that an electron can be added to the photoexcited dye in order to bring the dye back to the ground state. In the case of this solar cell, the mediator is the I^-^/I_3_^-^ redox couple, which has been shown to be the best known redox couple for this application [62]. The load to be driven by the solar cell is placed between the TCO and the counter electrode, which is on the opposite side of the solar cell from the TCO, and is generally covered by platinum. Platinum is considered due to its high electrocatalytic activity, which is necessary to reduce the redox electrolyte. This reduction of the redox electrolyte is necessary to return the oxidized redox electrolyte back to its normal state. The electrons that pass through the load and then end up on the platinum counter electrode are then transferred to the redox electrolyte which reduces the oxidized electrolyte [62].

The solar cell is assembled by dye sensitizing the TCO with the nanotube forest in a solution of the (Bu_4_N)_2_Ru(dcbpyH)_2_(NCS)_2_(N719) dye in dry ethanol. The platinum coated counter electrode, which is fabricated by evaporation onto an FTO electrode, and the dye-sensitized electrode are spaced apart with the use of hot melt spacers. The redox electrolyte (0.5 M LiI, 50 mM I_2_, 0.5 M 4-tertbutylpyridine in 3-methoxypropionitrile) is then added to the space between the two electrodes.

In order to optimize the performance of the nanotube forest as a DSSCs, the efficiency (η) and the fill factor (FF) should be well understood. The overall efficiency is important in determining the percentage of energy from the incident light that is able to be converted into useful electrical energy. The fill factor is used in determining how ideally the solar cell works as a diode, which can change depending on how well the counter electrode acts as an electrochemical catalyst, or how changing the electrolyte of the photovoltaic can affect the fill factor and be an indicator of how scalable the system is.

## Conclusion

The current research effort and developments made towards electronic device applications have been heavily influenced by the utilization of nanomaterials synthesized from biologically inspired templates. These fascinating developments were the direct result of the advantages of using biotemplates; specifically the capability to mass produce biomaterials of uniform size, precise structure, complex morphology, and surface modification functionality. Many of these properties enhanced the fabrication method of nanomaterials and their functionalities in electronic device components. Especially, it have been said that the current technological limitations of solar cells and batteries can be amended through the use of nanostructures due to their high surface area to volume ratio. Many biotemplates had successfully been used to fabricate nanomaterials for electronic devices. The favorable results from these works have inspired the possibility to use bacterial flagella as biotemplates. The bacterial flagella can be a viable choice for biotemplates because of their unique natural properties such as tubular structures with controllable length and their polymorphic transformation. Although comprehensive studies had been done on the synthesis of high quality nanomaterials using bacterial flagella biotemplates, research on using these flagella templated nanomaterial in practical application is still in an early stage. We believe that future direction of this work should be focused on applying them to electronic devices. To move towards to realization of this work, we must optimize the manufacturing process to generate large quantity of nanomaterials with high quality in more controllable, accruable, and simple ways. Therefore, the flagella biological properties combined with synthetic chemistry have promising prospect in the fabrication of useful materials for electronic devices with high yield and functionality.

## References

[1] Lee SW, Mao C, Flynn CE, Belcher AM (2002). Science.

[2] Knez M, Sumser M, Bittner AM, Wege C, Jeske H, Martin TP, Kern K (2004). Adv. Funct. Mater..

[3] Mao C, Solis DJ, Reiss BD, Kottmann ST, Sweeney RY, Hayhurst A, Georgiou G, Iverson B, Belcher AM (2004). Science.

[4] Stanca SE, Eritja R, Fitzmaurice D (2006). Faraday Discuss.

[5] Flynn CE, Lee S-W, Peelle BR, Belcher AM (2003). Acta Mater..

[6] Jo W, Freedman KJ, Kim MJ (2012). Mater. Sci. Eng. C.

[7] Hesse WR, Luo L, Zhang G, Mulero R, Cho J, Kim MJ (2009). Mater. Sci. Eng. C.

[8] Atsumi T, Theor J (2001). Biol..

[9] Gekko K, Hasegawa Y (1986). Biochemistry.

[10] Oosawa F, Fujime S, Ishiwata SI, Mihashi K (1973). Cold Spring Harb. Sym..

[11] Kamiya R, Asakura S, Mol J (1976). Biol..

[12] Kamiya R, Asakura S, Mol J (1976). Biol..

[13] Asakura S (1970). Adv. Biophys..

[14] Yonekura K, Maki-Yonekura S, Namba K (2003). Nature.

[15] NAMBA K, VONDERVISZT F, Rev Q (1997). Biophys.

[16] Samatey FA, Imada K, Nagashima S, Vonderviszt F, Kumasaka T, Yamamoto M, Namba K (2001). Nature.

[17] Malapaka RRV, Adebayo LO, Tripp BC, Mol J (2007). Biol..

[18] Calladine C (1975). Nature.

[19] Kamiya R, Asakura S, Wakabayashi K, Namba K, Mol J (1979). Biol..

[20] Arkhipov A, Freddolino PL, Imada K, Namba K, Schulten K (2006). Biophys. J..

[21] Hasegawa K, Yamashita I, Namba K (1998). Biophys. J..

[22] Darnton NC, Berg HC (2007). Biophys. J..

[23] Kamiya R, Asakura S, Yamaguchi S (1980). Nature.

[24] Hasegawa E, Kamiya R, Asakura S, Mol J (1982). Biol..

[25] Kamiya R, Hotani H, Asakura S (1982). Symp. Soc. Exp. Biol..

[26] Washizu M, Shikida M, Aizawa S-I, Hotani H (1992). IEEE Ind. Applic. Soc..

[27] Jo W, Freedman KJ, Yi DK, Kim MJ (2012). Nanotechnology.

[28] Han Z, Tongxiang F, Ting H, Xufan L, Jian D, Di Z, Qixin G, Hiroshi O (2009). Nanotechnology.

[29] Davis SA, Burkett SL, Mendelson NH, Mann S (1997). Nature.

[30] Shenton W, Douglas T, Young M, Stubbs G, Mann S (1999). Adv. Mater..

[31] Huang Y, Chiang C-Y, Lee SK, Gao Y, Hu EL, Yoreo JD, Belcher AM (2005). Nano Lett..

[32] Tseng RJ, Tsai C, Ma L, Ouyang J, Ozkan CS, Yang Y (2006). Nat. Nano.

[33] Jeffryes C, Gutu T, Jiao J, Rorrer GL, Mater J (2008). Res..

[34] Fang Y, Wu Q, Dickerson MB, Cai Y, Shian S, Berrigan JD, Poulsen N, Kröger N, Sandhage KH (2009). Chem. Mater..

[35] Huang WW, Wang ZL (2006). Nano Lett.

[36] Wang Z, Di Z, Tongxiang F, Jian D, Qixin G, Hiroshi O (2006). Nanotechnology.

[37] Gratzel M (2001). Nature.

[38] Mor GK, Shankar K, Paulose M, Varghese OK, Grimes CA (2005). Nano Lett..

[39] Wang Z-S, Kawauchi H, Kashima T, Arakawa H (2004). Coordin. Chem. Rev..

[40] Zhang W, Zhang D, Fan T, Gu J, Ding J, Wang H, Guo Q, Ogawa H (2008). Chem. Mater..

[41] Law M, Greene LE, Johnson JC, Saykally R, Yang P (2005). Nat. Mater..

[42] Macák JM, Tsuchiya H, Schmuki P (2005). Angew. Chem. Int. Edit..

[43] Jingbin H, Fengru F, Chen X, Shisheng L, Min W, Xue D, Zhong Lin W (2010). Nanotechnology.

[44] Martinson ABF, Elam JW, Hupp JT, Pellin MJ (2007). Nano Lett..

[45] Flynn CE, Mao C, Hayhurst A, Williams JL, Georgiou G, Iverson B, Belcher AM, Mater J (2003). Chem..

[46] Dang X, Yi H, Ham MH, Qi J, Yun DS, Ladewski R, Strano MS, Hammond PT, Belcher AM (2011). Nat. Nano.

[47] Lee YM, Kim YH, Lee JH, Park JH, Park N-G, Choe W-S, Ko MJ, Yoo PJ (2011). Adv. Funct. Mater..

[48] Jeffryes C, Campbell J, Li H, Jiao J, Rorrer G (2011). Energ. Environ. Sci..

[49] Heilman BD, Miaoulis IN (1994). Appl. Opt.

[50] Potyrailo RA, Ghiradella H, Vertiatchikh A, Dovidenko K, Cournoyer JR, Olson E (2007). Nat. Photon..

[51] Armand M, Tarascon JM (2008). Nature.

[52] Royston E, Ghosh A, Kofinas P, Harris MT, Culver JN (2007). Langmuir.

[53] Chen X, Gerasopoulos K, Guo J, Brown A, Wang C, Ghodssi R, Culver JN (2010). ACS. Nano.

[54] Pomerantseva E, Gerasopoulos K, Chen X, Rubloff G, Ghodssi R (2012). J. Power Sources.

[55] Nam KT, Kim D-W, Yoo PJ, Chiang C-Y, Meethong N, Hammond PT, Chiang Y-M, Belcher AM (2006). Science.

[56] Kang SM, Ryou M-H, Choi JW, Lee H (2012). Chem. Mater..

[57] Kumara MT, Tripp BC, Muralidharan S (2007). Chem. Mater..

[58] Deplanche K, Woods RD, Mikheenko IP, Sockett RE, Macaskie LE (2008). Biotechnol. Bioeng..

[59] Jo W, Darmawan M, Kim J, Ahn CW, Byun D, Baik SH, Kim MJ (2013). Nanotechnology.

[60] Diamandis E, Christopoulos T (1991). Clin. Chem..

[61] Cheang UK, Roy D, Lee JH, Kim MJ (2010). Appl. Phys. Lett..

[62] Luque A, Hegedus S (2011). Book Handbook of Photovoltaic Science and Engineering.

